# Refined *in vivo* model for bone regeneration: insights into scaffold architecture and porosity

**DOI:** 10.3389/fbioe.2026.1725958

**Published:** 2026-02-18

**Authors:** Laurine Marger, Mélanie Freudenreich, Mustapha Mekki, Daniel Manoil, Fabrice Marger, Sanae El Harane, Baptiste Charbonnier, Jérôme Charmet, Nicolo Brembilla, Olivier Preynat-Seauve, Stéphane Durual

**Affiliations:** 1 Laboratory of Biomaterials, Faculty of Medicine, University Clinics of Dental Medicine, University of Geneva, Genève, Switzerland; 2 Division of Cariology and Endodonty, Faculty of Medicine, University Clinics of Dental Medicine, University of Geneva, Genève, Switzerland; 3 Department of Medicine, Faculty of Medicine, University of Geneva, Genève, Switzerland; 4 Nantes Université, Oniris, INSERM, Regenerative Medicine and Skeleton, RMeS, UMR 1229, Nantes, France; 5 School of Engineering HE-Arc Ingénierie, HES-SO University of Applied Sciences Western Switzerland, Neuchâtel, Switzerland; 6 Division of Dermatology and Venereology, University Hospitals of Geneva, Geneva, Switzerland and HekeTiss, Geneva, Switzerland

**Keywords:** bone regeneration, *in vivo* model, osteoconduction, pore size, scaffold architecture

## Abstract

**Background:**

The architecture of bone substitute scaffolds—particularly pore size and organization—plays a crucial role in orchestrating immune responses, osteogenesis and angiogenesis. Yet, the mechanisms linking scaffold design to the temporal dynamics of bone regeneration remain partially understood. To address this, we established a refined *in vivo* model that integrates histological, molecular, and immunological analyses from a single explant, enabling spatially resolved insight into the bone healing process and dynamics.

**Methods:**

Using a dynamic rabbit calvarial model, we investigated 3D-printed calcium phosphate cement scaffolds designed with concomitant macroarchitectures of 250 μm and 500 µm pores within a single construct, allowing direct intra-animal comparison. The model recapitulated three vertically migrating zones of regeneration—regenerative, osteogenic, and granulation—captured at 2 and 4 weeks. Histomorphometric analyses quantified bone ingrowth, while laser microdissection enabled zone-specific transcriptomic profiling from paraffin-embedded sections previously used for (immuno-)histology. Gene expression was further validated by qPCR and complemented with immunohistochemical characterization of macrophage and neutrophil populations.

**Results:**

Histological analysis revealed a consistent spatial organization of bone regeneration across conditions. After 4 weeks, scaffolds with 250 µm pores exhibited more homogeneous and advanced bone formation than those with 500 µm pores or particulate substitutes. Transcriptomic analysis identified 280–381 differentially expressed genes between microporous architectures, with over half being non-coding RNAs, suggesting an important role for post-transcriptional regulation. Enrichment analyses indicated modulation of pathways involved in immune activity, ossification, calcium signaling and autophagy. Immunohistochemistry confirmed similar inflammatory mechanisms across both macroarchitectures but revealed earlier M1-to-M2 macrophage transition and faster inflammatory resolution with the finest porous network.

**Conclusion:**

This integrative *in vivo* model provides a robust workflow for correlating structural, cellular, and molecular dimensions of bone regeneration within the same specimen. The findings show that scaffold macroarchitecture influences both the extent and timing of immune and osteogenic processes. While scaffolds with 250 μm and 500 µm pores supported regeneration, the finer design consistently promoted more advanced tissue formation and maturation. These results underscore the key role of scaffold design in modulating bone healing and highlight this model as a platform for studying structure–function relationships in bone tissue engineering.

## 1 Introduction

Bone regeneration is a complex process that relies heavily on the architecture of biomaterial scaffolds, particularly pore size, interconnectivity, and geometry. Bioceramics such as hydroxyapatite (HA), tricalcium phosphate (TCP), and biphasic calcium phosphate (BCP) are widely used due to their osteoconductivity and compositional similarity to native bone mineral (Bose et al., 2012; Jeong et al., 2019; Lee et al., 2019; de Carvalho et al., 2024; Di Spirito et al., 2024). Their regenerative performance, however, depends strongly on porosity, which governs osteoblast adhesion, vascularization, nutrient diffusion, and mechanical stability. A minimum pore size of ∼100 µm is generally required to allow vascular invasion (Karageorgiou and Kaplan, 2005), and most studies converge on a favorable macropore range between 200 and 700 µm (Lee et al., 2019; Elsheikh et al., 2022; Yamahara et al., 2022; Mukasheva et al., 2024; Todd et al., 2024; Baggio et al., 2025). Within this window, several reports suggest that ∼500 µm pores promote more robust osteogenesis and angiogenesis compared to ∼250 µm (Lee et al., 2019; Deng et al., 2021; Yamahara et al., 2022; Toosi et al., 2023; Li X.-L. et al., 2025), but other investigations report effective outcomes across smaller or larger values depending on material, defect site, and biological model (Jeong et al., 2019; Yamahara et al., 2022; Zhou et al., 2023; de Carvalho et al., 2024; Mukasheva et al., 2024). This variability illustrates why, despite extensive research, a clear consensus on optimal porosity remains elusive.

Additive manufacturing of bioceramics has opened new perspectives by allowing precise control of pore size, shape, and interconnectivity. Preclinical studies with 3D-printed TCP/HA scaffolds have shown vertical bone ingrowth up to several millimeters in calvarial models, confirming the osteoconductive potential of regular architectures (Moussa et al., 2015; Carrel et al., 2016a; Carrel et al., 2016b; Liu et al., 2024). Clinical translation is emerging, with initial case reports indicating feasibility of printed calcium phosphate substitutes for ridge augmentation (Mangano et al., 2021; Perez et al., 2023; Schonegg et al., 2024). Alongside these, other groups have highlighted the advantages of triply periodic minimal surface (TPMS) structures, where gyroid and diamond geometries outperform primitive designs for vertical augmentation (Guerrero et al., 2023; Maevskaia et al., 2023; Maevskaia et al., 2024). Related works with lithography-based scaffolds also support the benefits of deterministic architectures for bone regeneration (Guerrero et al., 2023; de Carvalho et al., 2024). Compared to particulate substitutes such as DBBM or BCP granules, which present controlled microporosity but generate random intergranular voids and less predictable vertical stability (Fujioka-Kobayashi et al., 2021; Urban et al., 2021; Vaquette et al., 2021; Reich et al., 2022; Donos et al., 2023; Urban et al., 2023), printed scaffolds thus provide deterministic architectures, reliable space maintenance, and the possibility to optimize geometry at multiple scales. Nevertheless, even in these controlled systems, the debate remains unresolved regarding the superiority of ∼250 versus ∼500 µm pores (Krieghoff et al., 2019; Yamahara et al., 2022), reinforcing the need for standardized models and integrative analyses. Although bioactive scaffolds incorporating growth factors, peptides, or ions have been developed to enhance osteoinduction and angiogenesis (Szwed-Georgiou et al., 2023), this study deliberately focuses on architecture alone. By excluding additional bioactive cues, we aim to refine our understanding of how scaffold geometry intrinsically governs bone regeneration.

Beyond physico-chemical and architectural considerations, the biological mechanisms underlying scaffold-guided bone regeneration remain poorly understood. While osteogenesis and angiogenesis are recognized as central, the contributions of immune modulation, mechanotransduction, and dynamic cell–matrix signaling are only partially characterized (Dimitriou et al., 2011; Loi et al., 2016; Stewart et al., 2020; Ji et al., 2022; Su et al., 2022; Li X.-L. et al., 2025; Yang T. et al., 2025). Likewise, the temporal orchestration of immune responses—sometimes referred to as “immune choreography”—appears to influence scaffold integration and vascularization, particularly through macrophage polarization and cytokine signaling (Li J. et al., 2025). Emerging evidence also indicates that autophagy contributes to osteoblast differentiation and matrix remodeling, acting as a regulatory interface between mechanical stimuli and metabolic adaptation (Wu Y. et al., 2024). Although the relative weight of these pathways remains to be fully clarified, they provide a mechanistic framework through which scaffold geometry could shape the biological outcome of bone regeneration.

Omics-based approaches, including transcriptomic profiling, have been proposed to complement histology and morphometry, offering molecular insight into the pathways activated by specific scaffold designs (Ji et al., 2022; Guerrero et al., 2023; Wu J. et al., 2024; Yang L. et al., 2025). However, translating these molecular findings into robust preclinical evidence requires experimental designs that minimize biological variability. In this context, most available studies still test one pore size or architecture in separate animals, complicating direct comparisons due to inter-individual differences. This limitation underscores the importance of developing models that allow intra-animal comparisons, thereby strengthening statistical robustness while adhering to the 3R principles (Marger et al., 2019; Durual et al., 2021; Vaquette et al., 2021).

In this context, further refinements of established calvarial models integrating multiple pore architectures within the same scaffold and allowing multimodal analysis of a single explant represent a promising approach to generate new mechanistic insights and to contribute to the ongoing debate on optimal porosity and scaffold design for vertical bone regeneration.

## 2 Materials and methods

### 2.1 Scaffold production and devices

#### 2.1.1 3D-CPC scaffolds

**FIGURE 1:**
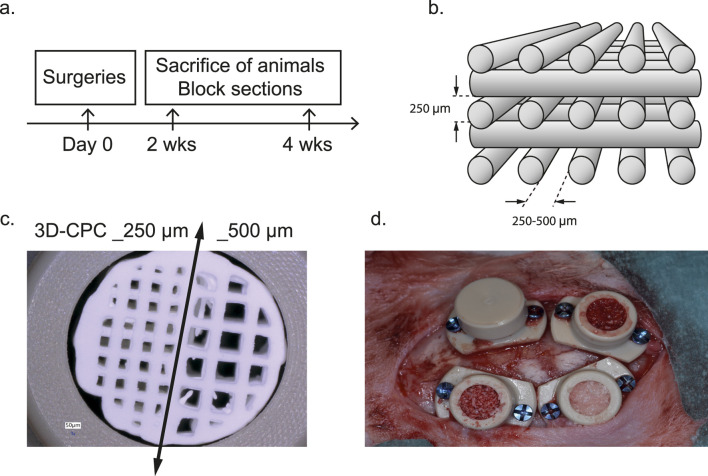
Study overview: **(a)** Time frame of the pre-clinical study. **(b)** Schematic representation of a 3D-printed scaffold with a layer-by-layer orthogonal architecture and macroporosity ranging from 250 to 500 µm. **(c)** Photograph of a combinatorial 3D-CPC scaffold with a macroporosity of 250 µm (left) and 500 µm (right), placed in a calvarial cylinder. **(d)** Illustration of the placement of four PEEK cylinders on the calvarium of a rabbit. The cylinders are filled with different substitutes and then closed, as shown in the top-left cylinder.

Scaffolds were designed using BioCAD software (RegenHu, Villaz-St-Pierre, Switzerland) (Figure 1b) and fabricated with an α-TCP/HA cement paste (Plotter-Paste-CPC, Innoterre GmbH, Radebeul, Germany) (Figure 1c). These cements yield scaffolds with well-characterized biological and mechanical properties and are CE-marked for maxillofacial applications by Innoterre GmbH (Moussa et al., 2015; Carrel et al., 2016a; Carrel et al., 2016b; Durual et al., 2021; Perez et al., 2023). Two distinct pore architectures were integrated within a single combinatorial scaffold, composed of two equal regions with rectilinear pores of (a) 250 µm and (b) 500 μm, separated by a non-porous wall. Each scaffold had a final diameter of 4.7 mm and a height of 5 mm, optimized to fit within PEEK cylinders. A series of scaffolds with uniform rectilinear porosity (250 or 500 µm) was also fabricated, specifically for the second round of surgeries and the subsequent qPCR analyses. Printing was performed via extrusion using a 3D Discovery printer (RegenHu) equipped with a 0.2 mm plastic needle nozzle, operated at room temperature under pressures of 0.2–0.4 MPa and a printing speed of 10 mm/s.

Scaffolds were fabricated in a layer-by-layer fashion, with orthogonally oriented rods of 250 µm in diameter. Post-printing solidification was carried out as follows: (1) drying for 5 days at 50 °C in a desiccator; (2) setting maturation in 0.9% NaCl solution for 2 days with regular solution replacement; (3) three sequential acetone washes i- to remove all trace of water and ii- to eliminate any trace of residual organic compounds added to improve the paste printability (20 min each); (4) air drying at room temperature for 12 h; and (5) gamma sterilization.

The morphological integrity and dimensional accuracy of the scaffolds were verified using an optical microscope (Keyence VHX-5000).

#### 2.1.2 P-CPC particles

All scaffolds that did not exhibit complete structural integrity after printing were ground into powder and sieved through a series of meshes (1 mm, 800 μm, 650 μm, 400 μm, and 250 µm). This process yielded CPC particles with irregular shapes and sizes ranging from 250 to 1,000 μm, comparable to the reference material Geistlich Bio-Oss® (GBO).

#### 2.1.3 Xenogenic bone particles GBO

Commercial bovine bone substitute particles (Geistlich Bio-Oss®, 250–1,000 μm; Geistlich Pharma AG, Wolhusen, Switzerland) were used as a xenogenic reference material.

#### 2.1.4 Cylindrical devices

Polyetheretherketone (PEEK) cylinders (Boutiplast, Leyment, France) with an inner diameter of 5 mm and a height of 5 mm were used as carriers, following previous studies (Marger et al., 2019).

### 2.2 Rabbit calvarial model and surgical procedure

**FIGURE 2:**
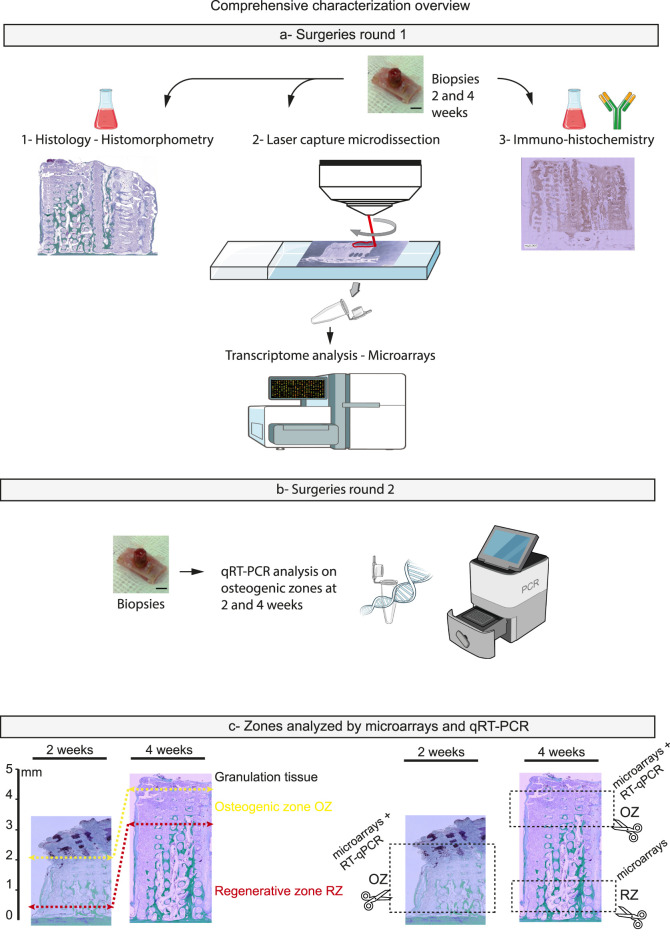
Figure illustrating the comprehensive characterization of biopsies obtained during preclinical phases. In round 1 **(a)** each biopsy was fixed and embedded in paraffin to enable histological staining (Masson–Goldner) and histomorphometric assessment of bone growth. Unstained paraffin sections from the same biopsies were subsequently microdissected by laser based on the histological results. Osteogenic regions (2–4 weeks) and regenerative regions (4 weeks), as depicted in section c, were isolated, and total RNA was extracted for transcriptomic analysis (microarray). Based on these findings, the same biopsies were further analyzed by immunohistochemistry. In a second surgical round **(b)** biopsies were directly sectioned according to the growth kinetics described in round 1. Osteogenic regions at 2 and 4 weeks were analyzed by qRT-PCR on a selected panel of genes related to the GO terms and KEGG pathways highlighted by the previous transcriptomic analysis.

All animal procedures were approved by the local ethics committee and conducted in compliance with cantonal and federal veterinary regulations (authorization numbers GE127-34014 and GE127A-34014).¬ A total of 6 male and 6 female New Zealand White rabbits (2.5–3.0 kg, aged over 3 months; UNIGE breeding facility, Arare, Switzerland) were used in the study. Two series of bone augmentation surgeries were carried out as previously described (Marger et al., 2019). Briefly, anesthesia was induced via intravenous injection of 2% propofol (Braun, Sempach, Switzerland) and maintained under 3% sevoflurane (AbbVie, Chicago, IL, USA) following intubation. Continuous intravenous infusion of remifentanil (0.008–0.5 μg/kg/min, 5 μg/mL; Bichsel, Unterseen, Switzerland) was administered through the ear vein to ensure intraoperative analgesia. Animals were positioned prone, and a midsagittal incision was made. After elevation of the periosteum and saline irrigation, four PEEK cylinders were placed and secured to the calvaria using CpTi Gr5 titanium microscrews (Global D, Brignais, France). Within each cylinder’s bone bed, five intramedullary holes (0.8 mm in diameter, 1 mm deep) were drilled to facilitate blood and cell infiltration. 3D-CPC Scaffolds, CPC particles, or GBO were randomly assigned to each cylinder according to a predefined experimental plan (Figure 1d). The quantities of CPC and GBO particles, as well as their preparation prior to insertion into the cylinders, were standardized. Each cylinder was therefore filled with identical amounts and underwent the same impregnation procedure. As a result, compaction and porosity were consistent across all cylinders. This procedure had been previously validated (Durual et al., 2021). The cylinders were sealed with PEEK caps, and the surgical site was closed using interrupted non-resorbable sutures (Prolene 4.0, Ethicon, Somerville, NJ, USA). Postoperative analgesia was provided with buprenorphine hydrochloride (Reckitt Benckiser, Slough, UK) administered four times daily for 3 days. Sutures were removed after 10 days, and the animals were monitored daily. Euthanasia was performed at 2 and 4 weeks post-surgery by overdose of pentobarbital following anesthesia (Intravenous injection of pentobarbital at a dose of 150 μg/kg (100 mg/mL), following a 2 mL bolus of remifentanil (10 µg)). For histological, immunohistochemical, and laser microdissection analyses (Round 1; Figure 2a), biopsies were fixed in 4% formalin, rinsed in PBS for 24 h, decalcified in Osteosoft (Sigma, St. Louis, MO, USA) for 3 weeks, and embedded in paraffin. This first round included 8 rabbits (4 sacrificed at 2 weeks and 4 at 4 weeks; n = 8 per sample). In the second series of surgeries (Round 2; Figure 2b, lower section), 4 rabbits were used (2 at 2 weeks and 2 at 4 weeks; n = 4 per sample). Biopsies from this group were dedicated to qRT-PCR analysis of osteogenic zones and were snap-frozen in RNA-free tubes at −80 °C until processing.

### 2.3 Histology and histomorphometry

The paraffin-embedded biopsy samples were sectioned at a thickness of 5 μm, deparaffinized, and stained with Masson–Goldner trichrome to visualize newly formed bone tissue. For each scaffold design, eight samples (n = 8) were analyzed. To assess the spatial distribution and uniformity of bone formation throughout the scaffold height, three distinct section levels were prepared from each sample. Comparison among the different section levels enabled evaluation of the consistency of bone regeneration across the scaffold depth. It was shown previously that the variation in bone regeneration was negligible across the different section levels (Marger et al., 2019; Durual et al., 2021; Marger et al., 2022).

Semi-quantitative analysis of newly formed bone was conducted on Masson–Goldner trichrome–stained sections by detecting the green-stained mineralized tissue, corresponding to newly formed bone. Pixel-level detection of the green signal was carried out using the optical imaging system (VHX-5000, Keyence, Itasca, IL, USA), which automatically quantified the area (mm^2^) of the green-labeled regions. To correct for potential variability in scaffold occupancy within histological sections, all measurements were normalized to the available void area, defined as the total cross-sectional scaffold area excluding regions physically occupied by the material. This normalization ensured that bone formation was evaluated relative to the actual space available for tissue ingrowth.

Prior to analysis, all selected sections were screened to confirm that the scaffold occupied a comparable proportion of the analyzed region and did not excessively obscure the field of view, thereby minimizing inter-sample bias.

### 2.4 Immunohistochemistry and image analysis

Arg-1 (anti-hepatic arginase antibody, Abcam Cambridge UK, ab92274), iNOS (anti-iNOS antibody, EPR16635, Abcam, ab210823), and the T/neutrophil markers RPN3-57 (sc-59376, Santa Cruz Biotechnology, Heidelberg, Germany) were utilised to stain M2-type macrophages (anti-inflammatory response), M1-type macrophages (pro-inflammatory response) and neutrophils and T cells, respectively. Sections (5 µm) underwent antigen retrieval in 10 mM citrate buffer (pH 9; Dako s2367) for 10 min in a microwave oven. Following endogenous enzyme blocking (Dako k4065, 8 min), primary antibodies (dilution 1:200) were applied for 1 h. After rinsing, sections were incubated with HRP-labelled polymer (Dako EnVision + Dual Link System-HRP, k4065) for 30 min, followed by DAB+ and hematoxylin counterstaining. Immunostained sections were imaged at ×700 magnification using an optical microscope (VHX-5000, Keyence, Itasca, IL, USA). Quantitative analyses were performed to compare two timepoints (2 and 4 weeks) and two scaffold architectures (pattern a and b). For each condition and timepoint (n = 8), two slides were systematically analyzed by two independent operators. Each experiment was independently repeated three times on three consecutive ribbon sections.

Cell quantification was conducted using QuPath 0.5.1, an open-source digital pathology software (Bankhead et al., 2017). Positive cells were manually annotated for each marker, and their spatial distribution relative to the bone bed was computed. The bone bed was systematically annotated on each section and served as a reference baseline for all distance measurements. In total, 1,655 Arg-1–positive cells, 5,281 iNOS-positive cells, and 8,960 RPN3-57–positive cells were manually annotated across all samples. These data were compiled and processed using a custom Matlab-based program (Matlab R2021a, MathWorks, Natick, MA, USA), which generated distribution profiles according to the vertical axis of the scaffold, from the bone bed to the top of the PEEK cylinder. The resulting spatial distribution profiles are presented in Figures 8–10, superimposed on corresponding histological sections to illustrate the spatial distribution and organization of immune cells in each scaffold architecture.

### 2.5 Laser microdissection-transcriptome analysis- microarrays

From the same paraffin-embedded biopsy blocks used for histological analysis, 10 µm sections were mounted on PET-membrane slides (Leica, Thermo Fisher 11532325) and microdissected under a microscope (LMD 6500, Leica) at ×5 magnification. A total of eight independent samples (n = 8) were analyzed, with each sample processed on at least three consecutive serial ribbon sections to ensure representative and reproducible tissue collection. At 2 weeks, only the osteogenic zone was identified, whereas at 4 weeks, two distinct zones were selected: (1) mineralized bone adjacent to the bed and (2) the upper mineralizing (regenerative) zone located beneath the migration front (Figure 2). In each area, multiple circular regions (1,000 µm diameter) or equivalent surfaces were delineated, laser-cut and collected by gravity into RNAse-free tube caps. The collected paraffin-embedded biopsy fragments were subsequently processed for total RNA extraction using the MagMax FFPE DNA/RNA Ultra Kit (Thermo Fisher Scientific, Reinach, Switzerland). For quantitative and qualitative reasons (RIN 4–6), we pooled the samples from 8 biopsies for each condition. This resulted in 6 groups:

**Table:** 

​	Osteogenic 2 weeks	Osteogenic 4 weeks	Regenerative 4 weeks
3D-CPC 250	RNA from a pull of 8 biopsies	RNA from a pull of 8 biopsies	RNA from a pull of 8 biopsies
3D-CPC 500	RNA from a pull of 8 biopsies	RNA from a pull of 8 biopsies	RNA from a pull of 8 biopsies

A total of 50 ng of total RNA was used as input for target preparation using the GeneChip WT Pico Reagent Kit (ThermoFisher Scientific). Hybridization was performed on Affymetrix GeneChip Rabbit Gene 1.0 ST arrays, followed by washing, staining, and scanning with the Affymetrix system according to the manufacturer’s instructions. Gene annotation and functional enrichment analyses among significantly regulated transcripts (fold change >2 or < −2; FDR <0.01) were conducted using the Metascape online platform (Zhou et al., 2019), referencing the Gene Ontology (GO) Biological Process and Kyoto Encyclopedia of Genes and Genomes (KEGG) pathway databases. Principal Component Analysis (PCA) was performed with TAC 4.0.1.36 software (Biosystems, Muttenz, Switzerland) under default parameters. Gene Set Enrichment Analysis (GSEA) was applied to examine differential gene expression profiles between 3D-CPC scaffolds with pore sizes of 250 μm and 500 µm in the osteogenic zone at 2 and 4 weeks. GO enrichment analysis was used to classify differentially expressed genes according to their associated biological processes, molecular functions, and cellular components, while KEGG pathway analysis was applied to identify the principal signaling pathways involved.

### 2.6 qRT-PCR analysis

Total RNA was extracted from Round 2 biopsies using TRIzol Reagent (Thermo Fisher). RNA concentration and purity were determined by absorbance (260 nm and 260/280 ratio) using a Qubit 2.0 fluorometer (Thermo Fisher). cDNA synthesis was performed with Superscript II (Thermo Fisher). Quantitative real-time PCR (qRT-PCR) was conducted using TaqMan primers (ThermoFisher) targeting inflammatory, regenerative, and autophagy-related genes, includingIL1β (Oc03823250_s1), IL6 (Oc04097053_m1), TNFα (Oc03397715_m1), CXCL12 (Oc06731048_m1), MPO (Oc06780710_m1), ALOX15 (Oc03823548_s1), CCL2 (Oc03823583_s1), ARG1 (Oc03397218_m1), MAP1LC3C (Oc06779063_s1), ATG5 (Oc06724789_m1), ATG7 (Oc06724789_m1), WIPI1 (Oc06736980_m1), ULK1 (Oc06733082_g1), TLR4 (Oc03398504_m1), CD36 (Oc03395926_m1), PECAM1 (Oc06726473_m1), IL10 (Oc03396940_m1), SLC15A2 (Oc03398448), MMP12 (Oc03398612_m1), CXCL10 (Oc06781608_g1), FUT4 (Oc06805515_s1). GAPDH (Oc03823402_g1) served as the housekeeping gene. All reactions were performed in triplicate on a StepOne Plus thermal cycler (Applied Biosystems) under standard cycling conditions, and relative gene expression was quantified using the comparative RQ method. (n = 4 per condition).

### 2.7 Statistical analysis

For Histomorphometry analysis, Data were analyzed using GraphPad Prism version 10.4.1 (GraphPad Software, San Diego, CA, USA). Normality of data distribution was assessed using the Shapiro–Wilk test. When data followed a normal distribution, one-way analysis of variance (ANOVA) was applied. Post hoc multiple comparisons were performed using Fisher’s Least Significant Difference (LSD) test, without correction. When data did not meet normality assumptions, the non-parametric Kruskal–Wallis test was used instead, followed by uncorrected Dunn’s post hoc test for group comparisons. A p-value of <0.05 was considered statistically significant (*p < 0.05, **p < 0.001).

For qPCR analysis, a Student’s t-test was conducted to compare the mean values between the two groups. Prior to performing the analysis, the assumption of normality was evaluated using the Shapiro–Wilk test, which confirmed that the data were approximately normally distributed. Consequently, the t-test was deemed appropriate for assessing whether a statistically significant difference existed between the group means.

## 3 Results

### 3.1 Clinical course

All rabbits survived both the surgery and the postoperative bone growth period, which lasted up to 4 weeks. No signs of infection or inflammation were observed at the implantation site, nor during biopsy analysis. After 2 weeks, the upper part of the biopsies was sometimes missing, as it remained fragile and had not yet been colonized by cells. By 4 weeks, the samples had become denser, indicating complete colonization of the scaffolds.

### 3.2 Bone growth - histomorphometry

The dynamic bone growth model employed in this study has been previously described (Marger et al., 2019; Durual et al., 2021; Marger et al., 2022). As healing progresses, three distinct tissue zones emerge, each arranged sequentially from the bone bed upward:

1. Regenerative Zone (RZ): Situated directly on the bone bed, this zone is responsible for bone formation and remodeling. Its thickness increases notably over time—from approximately 1 mm at 2 weeks to 3 mm at 4 weeks. Within this region, both newly formed and mature lamellar bone are present, although fewer osteogenic precursor cells are found compared to the zones above.
2. Osteogenic Zone (OZ): Located above the remodeling region, this area is highly vascularized, rich in osteoid tissue, and populated by numerous bone-forming precursor cells. Here, tissue mineralization begins, and osteoblasts actively produce new bone.
3. Granulation Zone (GT): Found at the uppermost level, this region consists of inflammatory cells, fibrous tissue, and new blood vessels. It gradually migrates upward over time—starting near the bone bed at 3 days, reaching the scaffold’s midsection by 2 weeks, and extending to the top by 4 weeks. This zone contains a low density of bone precursor cells.

As bone formation advances, these three zones shift upward, reflecting a dynamic pattern of bone growth governed by the scaffold’s structural design.

Using this model, our objective was to demonstrate the importance of controlling (i) scaffold architecture and (ii) pore size to optimize bone growth and osteoconduction. To achieve this, we compared 3D-printed calcium phosphate cement scaffolds (3D-CPC) with particulate substitutes composed either of bovine bone (GBO) or the same calcium phosphate cement used for the 3D-CPC (P-CPC). Each 3D-CPC scaffold incorporated two distinct orthogonal architectures within half of its volume, corresponding to pore sizes of 250 μm and 500 µm (Figures 1b,c). The particulate substitutes, by contrast, exhibited pore sizes ranging from 250 μm to 1,000 µm. All constructs had an overall porosity of approximately 60%.

**FIGURE 3:**
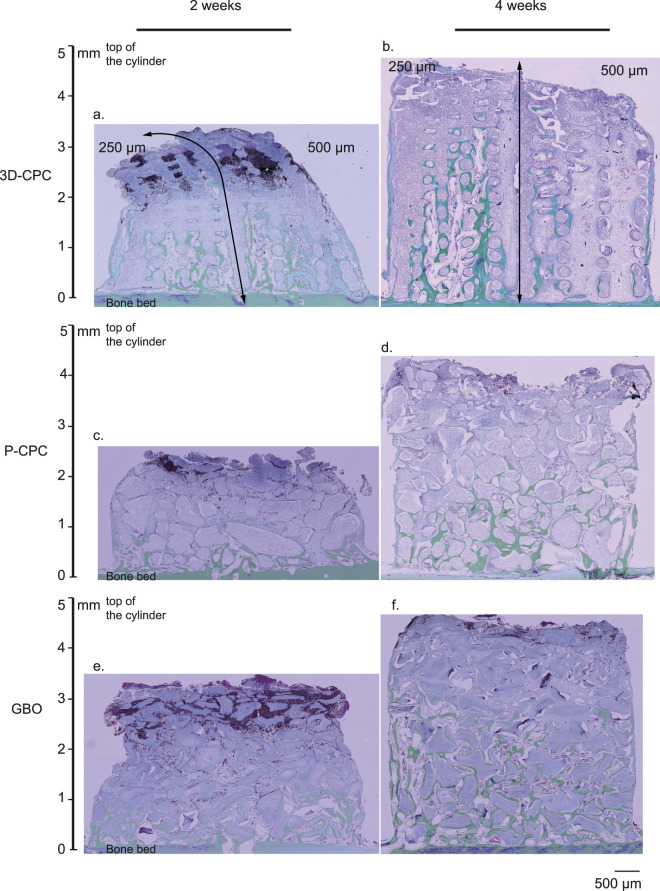
Representative images obtained at 2 and 4 weeks after the engraftment of the different bone substitutes within the cylinders, analyzed by histology (Masson-Goldner staining). **(a,b)** 3D-CPC scaffolds (marbled mauve). Two pore sizes are visible, 250 µm on the left and 500 µm on the right. Newly formed bone (green) originates from the bone bed and subsequently develops into vertical columns extending from the bony bed toward the top of the cylinders, particularly evident at 4 weeks in the 250 µm scaffold. Notably, horizontal (i.e., perpendicular) channels are filled with new bone, even at the upper part of the construct, and may contain a central vascular system. At 4 weeks, a difference in bone height can be observed within the scaffold: the 250 µm side appears to promote taller vertical bone growth than the 500 µm side. Finally, a loose, non-mineralized tissue rich in vasculature is consistently present adjacent to the newly formed bone on both architectures, at 2 and 4 weeks. **(c,d)** Particulate P-CPC scaffolds, and **(e,f)** GBO scaffolds, at 2 and 4 weeks. Marbled mauve particles are surrounded and bridged by newly formed bone, primarily in close proximity to the bone bed at 2 weeks, and extending further upward at 4 weeks. No clear difference in bone height was observed between these two conditions, which appear equivalent, though both were surpassed by the 3D-CPC 250 µm scaffold in terms of vertical bone ingrowth. As with 3D-CPC, in both conditions and especially at 4 weeks, a soft, highly vascularized tissue was present between the particles and the newly formed bone. Across all conditions, granulation tissue was evident at 2 weeks, extending up to ∼1 mm from the top of the scaffolds. At 4 weeks, this tissue persisted but was restricted to a very small area at the uppermost surface of the constructs. (3D-CPC, 3D printed calcium phosphate cement scaffold; P-CPC, particulate calcium phosphate cement scaffold; GBO, Geistlich Bio-oss®).

**FIGURE 4:**
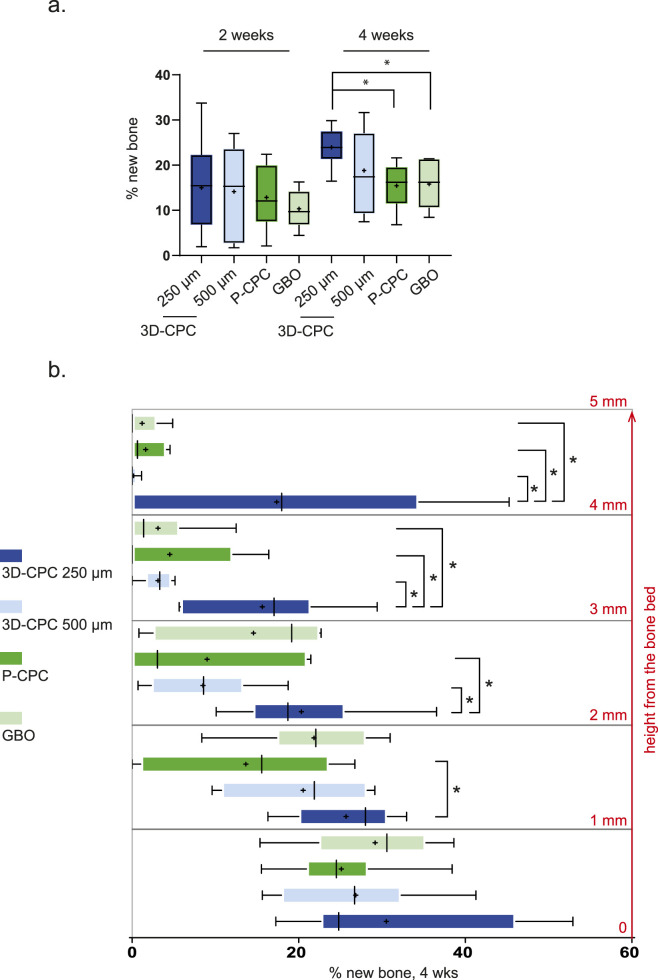
**(a)** Histomorphometric quantification of new bone formation (%) within 3D-CPC (250 and 500 µm), P-CPC, and GBO at 2 and 4 weeks (n = 8 per group). Brackets and * indicate statistically significant differences (p ≤ 0.05). Horizontal line: median; boxes: interquartile range (25%–75%); +: mean; whiskers: range of non-outliers. **(b)** Percentage of new bone volume within the bone substitutes, analyzed at 4 weeks across five horizontal planes of 1 mm in height (Cylinder 5 mm height). Brackets and * indicate statistically significant differences (p ≤ 0.05). Horizontal line: median; boxes: interquartile range (25%–75%); +: mean; whiskers: range of non-outliers. (3D-CPC, 3D printed calcium phosphate cement scaffold; P-CPC, particulate calcium phosphate cement scaffold; GBO, Geistlich Bio-oss®).

Across all conditions (3D-CPC_250–500 μm, P-CPC, and GBO), we observed consistent bone growth (Figure 3) that aligned perfectly with the aforementioned model (Marger et al., 2019; Durual et al., 2021; Marger et al., 2022). After 2 weeks, there were no significant differences in total bone growth, with the mean percentage of newly formed bone around 13% across all conditions (Figures 3, 4a). By 4 weeks, however, total bone filling varied depending on the condition. The 3D-CPC 250 µm scaffold exhibited a significantly higher filling rate than both P-CPC (15.5% ± 2.2%) and GBO (15.8% ± 2.3%). No significant difference was detected between the 3D-CPC 250 μm and 500 µm groups, which showed mean filling rates of 24% ± 1.6% and 19% ± 3.4%, respectively. It is worth noting that value dispersion was considerably greater in the 500 µm condition compared to the 250 µm condition (Figures 3, 4a).

A more detailed analysis enabled a precise evaluation of vertical bone growth. For this purpose, the measurement planes were divided into five consecutive 1 mm-thick layers starting from the bone bed (Figures 3, 4b).

At 2 weeks, no differences were observed between conditions, with mean filling rates of approximately 20% between 0 and 1 mm and 8% between 1 and 2 mm (data not shown). At 4 weeks, there were still no significant differences in the 0–1 mm layer, with an average bone filling of about 28% across all groups. However, between 1 and 2 mm, 3D-CPC 250 µm (25.7% ± 2.3% new bone) significantly outperformed P-CPC (13.7% ± 4.7% new bone). Between 2 and 3 mm, 3D-CPC 250 µm (19.4% ± 3.3% new bone) was superior to both P-CPC (9% ± 5%) and 3D-CPC 500 µm (11.7% ± 3.9%). Beyond 3 mm above the bone bed, 3D-CPC 250 µm continued to outperform all other conditions, maintaining bone filling levels of 13.4% ± 3.8% between 3 and 4 mm and 14.5% ± 7.5% between 4 and 5 mm, whereas the other bone substitutes, combined, showed filling values of 4.4% between 3 and 4 mm and 1.75% between 4 and 5 mm. No significant differences were ever observed among the 3D-CPC 500 μm, P-CPC, and GBO conditions.

### 3.3 Transcriptomic analysis

To elucidate the differences in bone growth and osteoconduction, we conducted a transcriptomic analysis. The dynamic bone growth model was particularly well-suited for this investigation, as all the distinct zones of bone regeneration coexist within each sample and migrate vertically over time. To streamline the study, we focused on the differential analysis between the 3D-CPC_250 μm and 3D-CPC_500 µm scaffolds.

We also sought to confirm that our model could be optimized in terms of biopsy processing. Accordingly, we used the same biopsies previously employed for (immuno-)histological analysis. Paraffin-embedded sections were microdissected for RNA extraction, with the osteogenic and regenerative zones identified histologically at 2 and 4 weeks, respectively.

**FIGURE 5:**
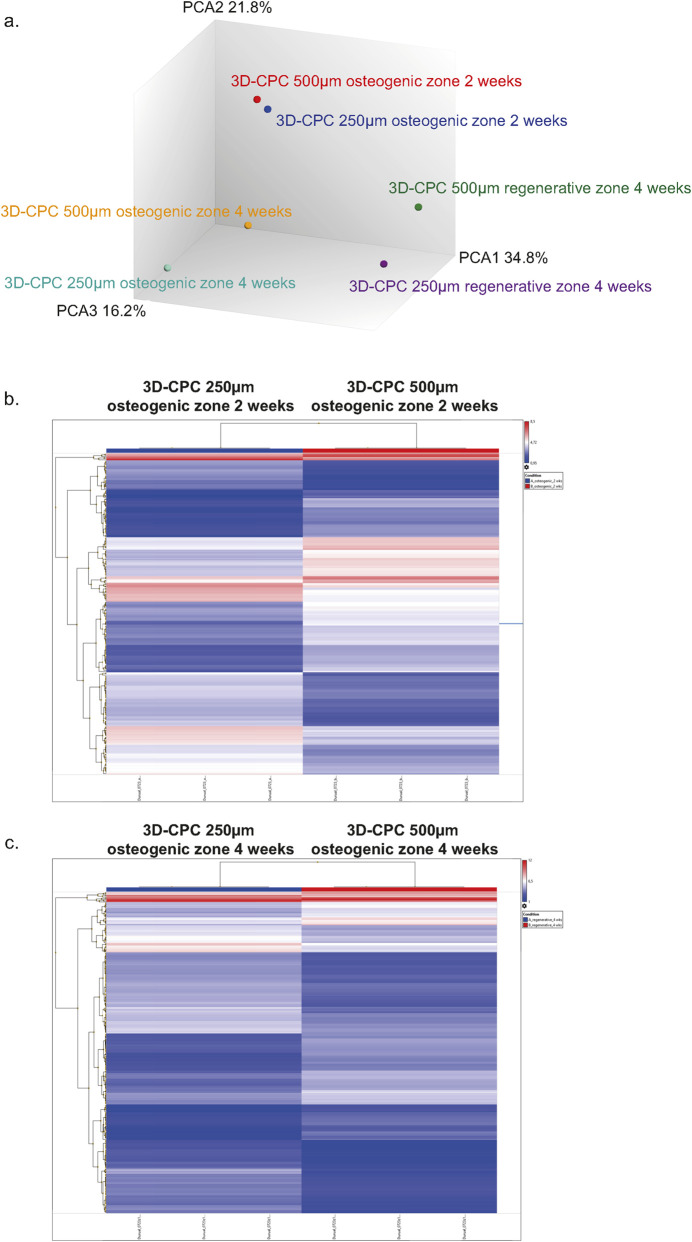
Microarray analysis of gene expression in 3D-CPC scaffolds with 250 μm and 500 µm pore sizes at 2 and 4 weeks **(a)** Principal Component Analysis. Each dot represents a pool of 8 biopsies from the osteogenic zones (2 and 4 weeks) or the regenerative zone (4 weeks). The percentages shown denote the fraction of total variance in the dataset explained by each principal component. **(b,c)** Heatmap of differential gene expression in the osteogenic zone at 2 and 4 weeks, comparing 250 μm and 500 µm scaffolds. Expression levels are color-coded to highlight temporal changes and pore-size–dependent differences.

Principal component analysis (PCA) of the transcriptomic data revealed distinct gene expression patterns between the 3D-CPC 250 μm and 500 µm conditions (fold change >2 or <0.5, p < 0.05). At 2 weeks, the two groups appeared relatively close, indicating only limited—but still significant—differences in the early activation of bone regeneration mechanisms (Figure 5a). Hierarchical clustering further confirmed distinct clusters of differentially expressed genes (Figure 5b). In total, 280 genes were identified as differentially expressed, with 134 upregulated and 146 downregulated. Notably, approximately 60% of these genes were non-coding RNAs, suggesting extensive post-transcriptional regulation.

By 4 weeks, the PCA data showed greater separation between the two conditions in both the osteogenic and regenerative zones, indicating that differences between the 3D-CPC 250 μm and 500 µm architectures became more pronounced over time (Figure 5a). Focusing on the osteogenic zone, hierarchical clustering (Figure 5c) confirmed the presence of distinct gene expression profiles. At this stage, 294 genes were differentially expressed (150 upregulated, 144 downregulated), with approximately 55% being non-coding RNAs, indicating sustained post-transcriptional regulatory activity. In the regenerative zone at 4 weeks, the data also showed considerable divergence, with 381 differentially expressed genes (222 upregulated, 159 downregulated), of which 53% were non-coding.

We then focused on the early activation of bone regeneration mechanisms, concentrating our analysis on the osteogenic zone at 2 and 4 weeks.

**FIGURE 6:**
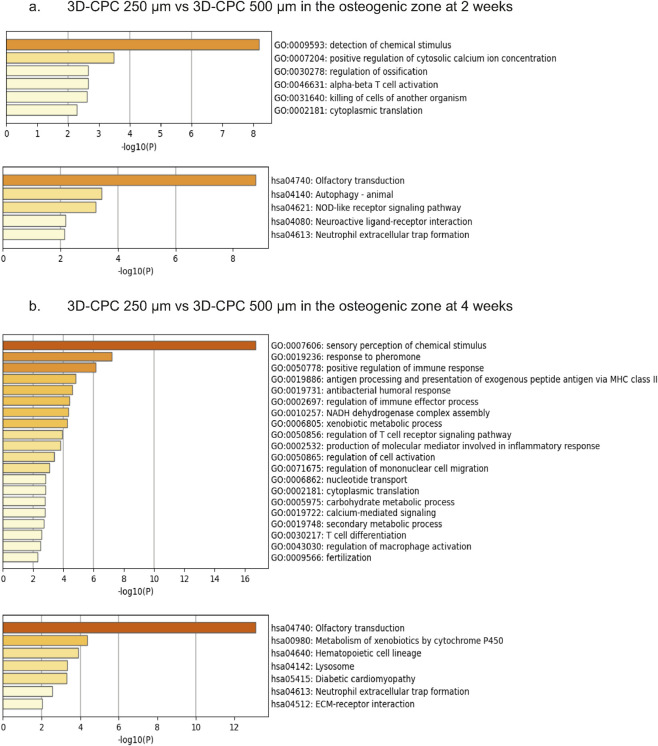
Differential gene expression analysis of 3D-CPC scaffolds with pore sizes of 250 μm and 500 µm in the osteogenic zone at 2 weeks **(a)** and 4 weeks **(b)**. n = 8 per sample. Gene Ontology (GO) enrichment analysis was performed to classify differentially expressed genes according to their associated biological processes, molecular functions, and cellular components. Kyoto Encyclopedia of Genes and Genomes (KEGG) pathway analysis was further applied to identify the main signaling pathways affected.

Using the online resource Metascape (Zhou et al., 2019), we performed gene annotation and statistical functional enrichment analyses based on the Gene Ontology (GO Biological Processes) and Kyoto Encyclopedia of Genes and Genomes (KEGG) databases. Among the most significantly enriched pathways differentially expressed between the 3D-CPC 250 μm and 500 µm conditions at 2 weeks were regulation of ossification, autophagy, and positive regulation of cytosolic calcium ion concentration, as well as several inflammation-related pathways, including neutrophil extracellular trap (NET) formation, NOD-like receptor signaling, and α-β T cell activation (Figure 6a).

At 4 weeks, numerous inflammatory pathways remained differentially expressed in the osteogenic zone, including NET formation, regulation of macrophage activation and mononuclear cell migration, regulation of immune response, and lymphocyte differentiation. Additionally, calcium signaling pathways and ECM–receptor interactions were notably enriched. As expected, several inflammatory pathways linked to the initiation of bone formation were identified. Interestingly, scaffold architecture appeared to modulate the activation intensity of these mechanisms. Moreover, the involvement of autophagy in early bone regeneration was particularly noteworthy, as it too seemed influenced by scaffold design (Figure 6b).

**FIGURE 7:**
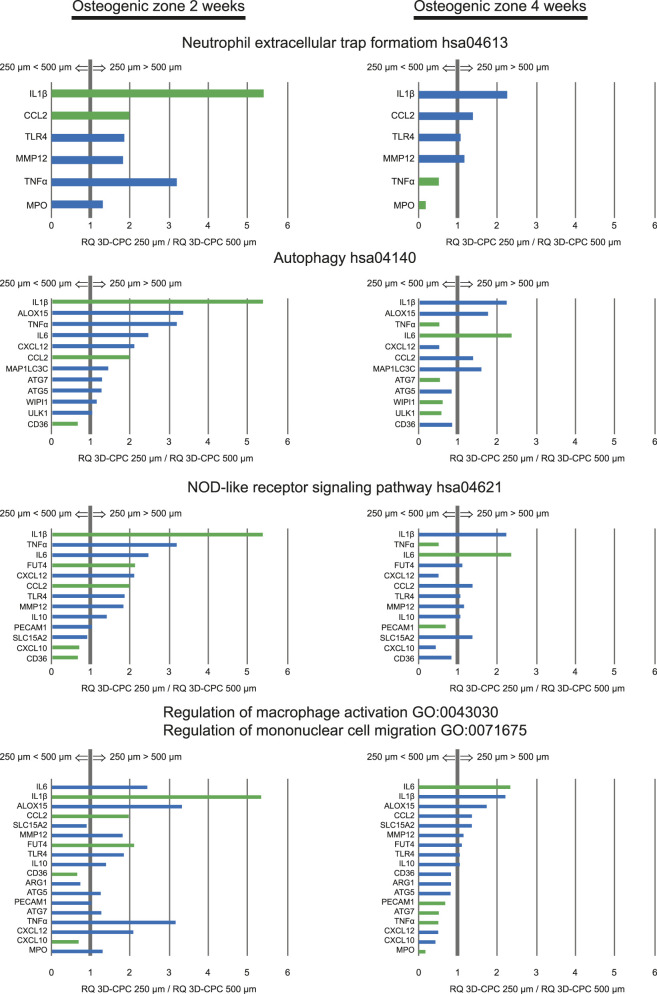
Differential expression of selected genes involved in immune and inflammatory pathways between 3D-CPC scaffolds with 250 μm and 500 µm pore sizes in the osteogenic zone, as assessed by quantitative RT-PCR. Gene expression ratios (RQ, 3D-CPC 250 µm/3D-CPC 500 µm) are shown for two time points: 2 weeks and 4 weeks. Genes associated with neutrophil extracellular trap formation (KEGG hsa04613), autophagy (KEGG hsa04140), and NOD-like receptor signaling (KEGG hsa04621) were analyzed, as well as those related to macrophage activation and mononuclear cell migration (GO:0043030, GO:0071675). This analysis highlights pore size–dependent modulation of immune-related pathways during osteogenesis. Green bars, significant difference, blue bars, tendency; p ≤ 0.05, n = 4 per condition.

In a subsequent series of experiments, scaffolds were again placed within the dynamic bone growth model, and biopsies were directly processed for total RNA extraction from the osteogenic zones at 2 and 4 weeks. We then performed qPCR analyses targeting a panel of genes involved in NET formation, autophagy, NOD-like receptor signaling, and macrophage activation and mononuclear cell migration (Figure 7). For example, IL1β, a pro-inflammatory cytokine involved in all these processes and a potent pro-angiogenic factor, was expressed approximately fivefold higher in the osteogenic zone of 3D-CPC_250 µm compared with 3D-CPC_500 μm at 2 weeks, a trend that persisted at 4 weeks. CCL2, a key mediator of autophagy, macrophage chemoattraction, and angiogenesis, showed about twofold higher expression at 2 weeks in 3D-CPC_250 µm than in 3D-CPC_500 µm. Conversely, ULK1 and WIPI1, major autophagy markers, exhibited a twofold decrease in expression in 3D-CPC_250 µm compared to 3D-CPC_500 μm at 4 weeks. Similarly, MPO and TNFα, strong indicators of NET formation, were markedly downregulated.

Overall, the analyzed gene panels showed statistically significant differential expression and clear trends consistent with the involvement of NET formation, autophagy, NOD-like receptor signaling, and macrophage activation and migration in the distinct bone growth responses induced by the two scaffold architectures, 3D-CPC_250 μm and 3D-CPC_500 µm. These targeted transcriptional analyses thus confirmed the findings of the initial enrichment analyses.

### 3.4 Immunohistochemical validation

Histomorphometric and transcriptomic analyses revealed clear differences in bone growth between the two adjacent architectures of the 3D-CPC scaffolds with pore sizes of 250 μm and 500 µm. These variations likely stem from distinct inflammatory mechanisms that initiate bone regeneration at different time points.

**FIGURE 8:**
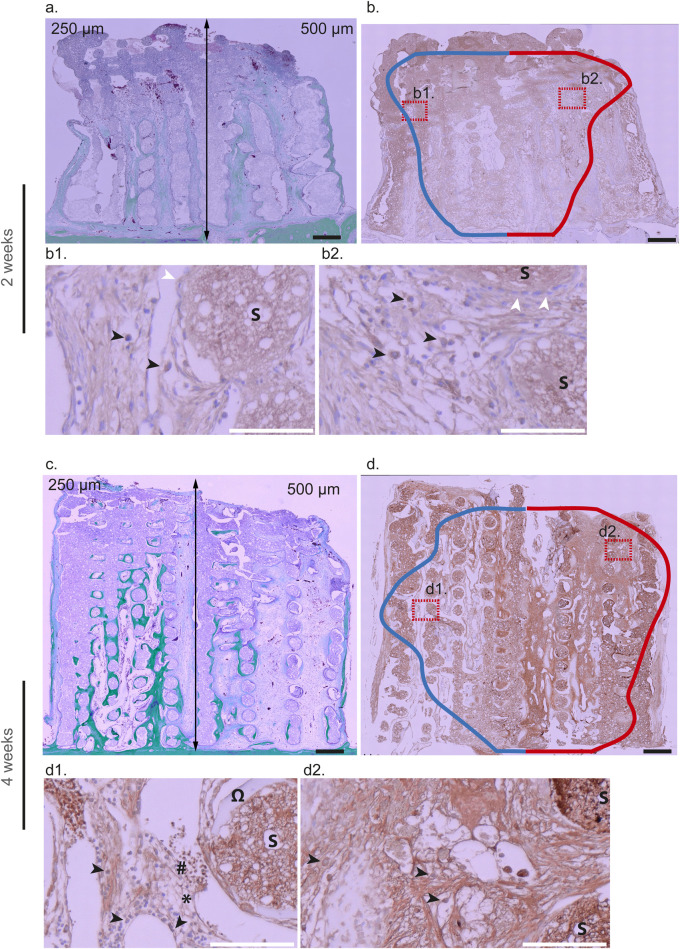
iNOS immunohistochemical analysis at 2 weeks **(a,b,**b1,b2**)** and 4 weeks **(c,d,**d1,d2**)** within 3D-CPC scaffolds (250 and 500 µm) M1 macrophages + neutrophils and lymphocytes staining. 2 Weeks: Representative image of a combinatorial scaffold stained with Masson-Goldner **(a)** showing bone formation (green) within pores of 250 µm (left) and 500 µm (right). The same biopsy was immunostained with an anti-iNOS antibody **(b)** and violin plot contours of iNOS-positive cell distribution (n = 8) were superimposed. The blue and red curves denote the distribution of iNOS-positive cells within scaffolds featuring pore sizes of 250 µm and 500 μm, respectively, whereas the horizontal dispersion represents the probability density of these cells occurrence as a function of distance (smoothed by a kernel density estimator). These analyses revealed that iNOS-expressing cells were predominantly macrophages located at the top of the scaffold, in close proximity to the granulation tissue, at the upper limit of the osteogenic zone. At higher magnification, these cells (black arrows) were observed within a loose, highly vascularized fibrous tissue where osteoblasts (white arrows) had begun to organize (b1: 250 μm; b2: 500 µm). 4 weeks: Representative Masson-Goldner staining of a combinatorial scaffold **(c)** illustrating bone formation (green) within 250 µm pores (left) and 500 µm pores (right). The corresponding biopsy was subsequently immunostained with an anti-iNOS antibody **(d)** and violin plot contours depicting the distribution of iNOS-positive cells (n = 8) were overlaid (blue line: 250 μm; red line: 500 µm). In 3D-CPC_250 µm scaffolds, staining was predominantly localized in the central region, where mature bone tissue (Ω) with marrow inclusions (*) containing myeloid (black arrows) and lymphoid cells (#) was observed (d1). In contrast, in 3D-CPC_500 µm scaffolds, staining was mainly concentrated at the scaffold top, within a dense, fibrous, and vascularized osteoid-like tissue enriched in macrophages (black arrows) (d2). The scaffold is tagged by a S. Black bars 500 μm, White bars 100 µm.

**FIGURE 9:**
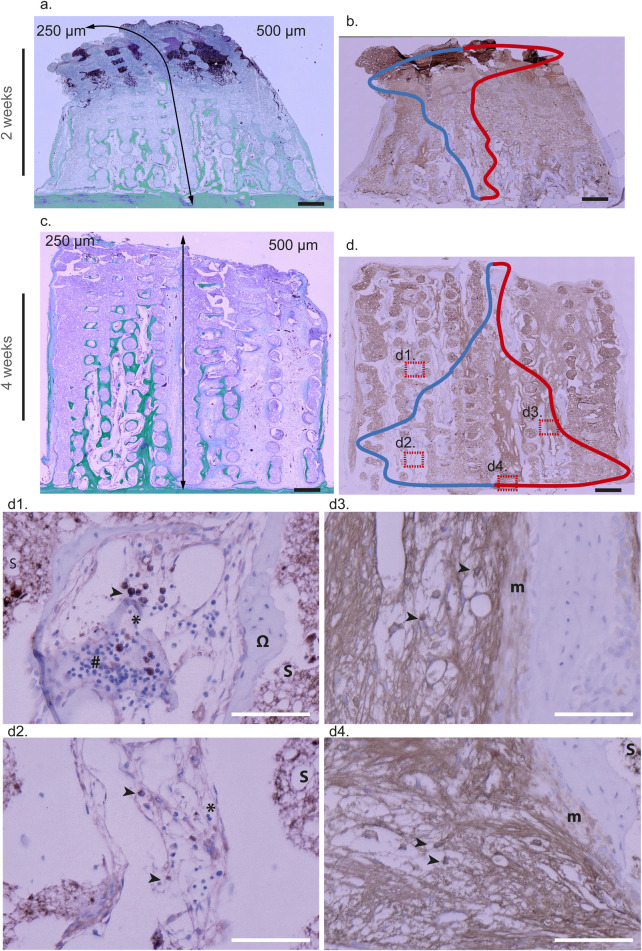
RPN3-57 immunohistochemical analysis at 2 weeks **(a,b)** and 4 weeks **(c, d,** d1–d4**)** within 3D-CPC scaffolds (250 and 500 µm) neutrophils and lymphocytes staining. 2 Weeks: representative image of a combinatorial scaffold stained with Masson–Goldner **(a)** illustrating bone formation (green) within pores of 250 µm (left) and 500 µm (right). The same biopsy was subsequently immunostained with the RPN3.57 antibody **(b)** and violin plot contours depicting the distribution of RPN3.57-positive cells (n = 8) were superimposed. The blue and red curves correspond to the distribution of RPN3-57-positive cells in scaffolds with pore sizes of 250 µm and 500 μm, respectively, while the horizontal spread illustrates the probability density of cell occurrence as a function of distance (smoothed by a kernel density estimator). Neutrophils and lymphocytes were abundantly present at the migration front in both the 3D-CPC_250 μm and 3D-CPC_500 µm scaffolds. 4 weeks: Masson–Goldner staining of a representative combinatorial scaffold **(c)** highlights bone formation within pores measuring 250 µm (left) and 500 µm (right). The matched biopsy was further processed for immunostaining using the RPN3.57 antibody **(d)**. Violin plot outlines illustrating the distribution of RPN3-57-positive cells (n = 8) were then overlaid (blue contour: 250 μm; red contour: 500 µm). Staining localized mainly to the central scaffold region near the bone bed. In 3D-CPC_250 µm scaffolds, mature mineralized bone (Ω) with marrow spaces (*) containing neutrophils (black arrows) and lymphocytes (#) was observed (d1,d2) whereas 3D-CPC_500 µm scaffolds showed less mature, fibrous, vascularized tissue undergoing mineralization (m) with frequent neutrophils (black arrows) (d3,d4). The scaffold is tagged by a S. Black bars 500 μm, White bars 100 µm.

**FIGURE 10:**
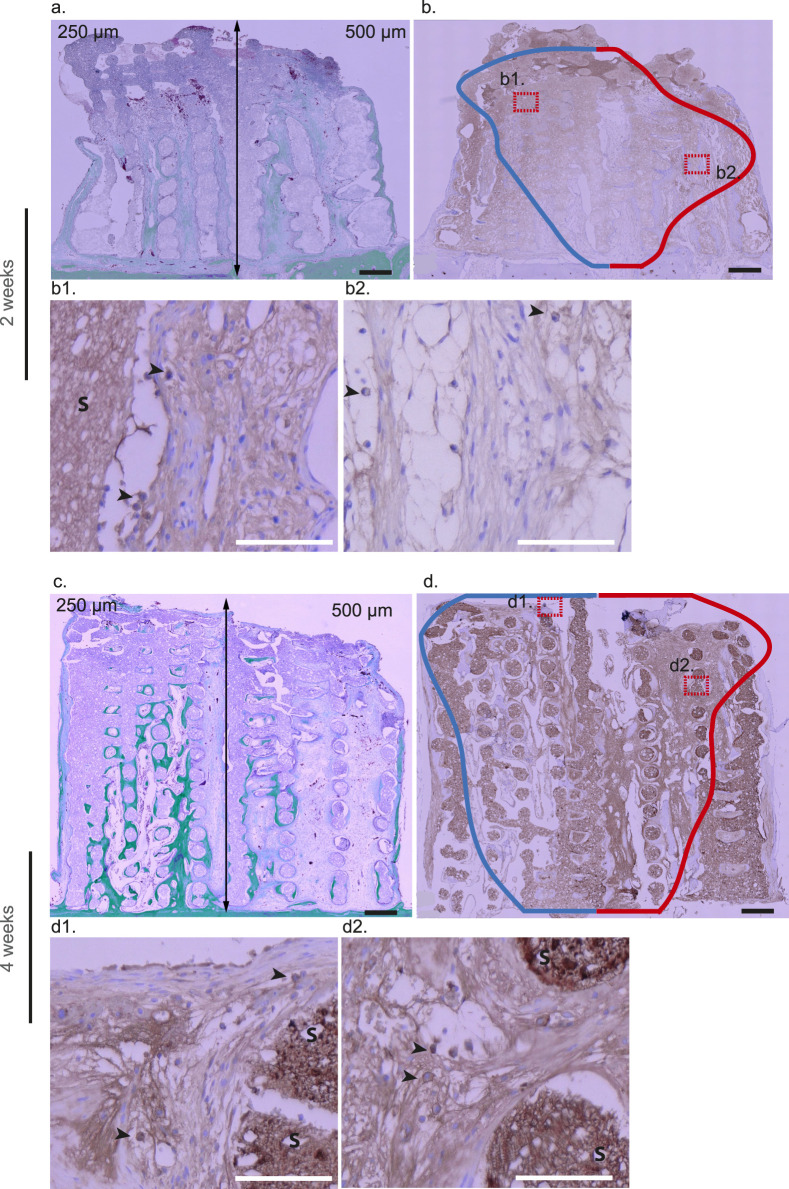
ARG1 immunohistochemical analysis at 2 weeks **(a,b,**b1,b2**)** and 4 weeks **(c, d,** d1,d2**)** within 3D-CPC scaffolds (250 and 500 µm) neutrophils and macrophages M2 staining. Masson–Goldner staining at 2 and 4 weeks **(a,c)** showed bone formation (green) within pores of 250 µm (left) and 500 µm (right). The same samples were then immunostained for ARG1 **(b,d)** and violin plots of ARG1-positive cell distribution were superimposed. The blue and red curves indicate the spatial distribution of ARG1-positive cells within scaffolds possessing pore diameters of 250 µm and 500 μm, respectively, while the horizontal profile reflects the probability density of cell occurrence across distances (smoothed by a kernel density estimator). (n = 8). At 2 weeks, ARG1-positive cells (black arrows) were mainly detected at the upper region of 250 µm scaffolds, close to the migration front, whereas in 500 µm scaffolds staining was more pronounced in central osteoid-rich areas. By 4 weeks, ARG1 expression shifted upward in both scaffold types, with M2 macrophages and neutrophils present within mineralizing tissue. In 500 µm scaffolds, this zone was located slightly deeper than in the 250 µm scaffolds. The scaffold is tagged by a S. Black bars 500 μm, White bars 100 µm.

To test this hypothesis, we performed immunohistological analyses on biopsies from the initial experimental series. At 2 and 4 weeks, M1 macrophages (and neutrophils) were identified using iNOS staining (Figure 8), neutrophils (and T lymphocytes) using RPN3-57 staining (Figure 9), and M2 macrophages (and neutrophils) using ARG1 staining (Figure 10).

Pro-inflammatory M1 macrophages are known to initiate bone regeneration mechanisms. Examination of their distribution in 2-week samples, through iNOS staining, revealed their presence throughout the sections from the bone bed upward, with a higher density near the migration front in both the 3D-CPC_250 μm and 500 µm architectures (Figures 8a,b). At higher magnification, these cells were observed within loose, highly vascularized fibrous tissue where osteoblasts had begun to organize (Figures 8b1,b2).

At 4 weeks, differences in iNOS staining patterns were apparent between the 3D-CPC_250 μm and 500 µm scaffolds. In the 3D-CPC_250 µm scaffolds, staining was more concentrated in the central region of the section (Figures 8c,d). At this stage, the bone tissue was mature, containing bone marrow inclusions rich in myeloid cells such as monocytes, macrophages, neutrophils, and lymphocytes (Figure 8d1). In contrast, in the 3D-CPC_500 µm scaffolds, staining remained strongest at the top of the scaffold (Figures 8c,d), corresponding to a region occupied mainly by macrophages within dense, fibrous, and vascularized osteoid-like tissue (Figure 8d2).

Similar patterns were observed with RPN3-57 staining, used to identify neutrophils and lymphocytes. As expected, at 2 weeks these cells were abundant at the migration front in both 3D-CPC_250 μm and 500 µm scaffolds (Figures 9a,b). After 4 weeks, the staining became localized primarily in the central region of the scaffolds, close to the bone bed. At higher magnification, within the 3D-CPC_250 µm scaffolds, staining was found in mature, mineralized bone tissue containing bone marrow inclusions rich in neutrophils and lymphocytes (Figures 9d1,d2). In contrast, the 3D-CPC_500 µm scaffolds exhibited less mature tissue characterized by dense, fibrous, vascularized, and partially mineralized areas where neutrophils were frequently present (Figures 9d3,d4).

At 2 weeks, ARG1 staining was markedly more abundant at the top of the 3D-CPC_250 µm scaffolds near the migration front (Figures 10a,b). In this area, an osteoid-like tissue was visible, containing M2 macrophages and neutrophils (Figure 10b1). In the 3D-CPC_500 µm scaffolds, ARG1 staining was more intense in the central region, corresponding to an osteoid-rich tissue infiltrated by M2 macrophages and neutrophils (Figures 10a,b,b2). By 4 weeks, the osteoid-rich region had shifted upward in both scaffolds, maintaining similar structural characteristics. In the 3D-CPC_250 µm scaffolds, M2 macrophages and neutrophils were detected within mineralizing tissue located at the upper extremity of the scaffold (Figures 10c,d,d1). A comparable tissue was observed in the 3D-CPC_500 µm scaffolds near the top as well, though approximately 1 mm lower than the corresponding region in the 3D-CPC_250 µm scaffolds (Figures 10c,d,d2).

Overall, both 3D-CPC scaffold architectures—250 µm and 500 µm—appeared capable of initiating and sustaining bone regeneration and subsequent maturation through histologically similar inflammatory mechanisms, but with differing temporal dynamics. The 3D-CPC_250 µm architecture promoted faster bone formation and earlier tissue maturation, following a sequential activation pattern involving M1 macrophages and neutrophils, subsequently transitioning to M2 macrophages. In contrast, the same process appeared delayed over the 4-week period in the 3D-CPC_500 µm scaffolds.

## 4 Discussion

### 4.1 A dynamic bone growth model as the analytical backbone

The dynamic bone growth model applied here—characterized by the sequential emergence and upward migration of the regenerative (RZ), osteogenic (OZ), and granulation (GZ) zones—served as the backbone for all analyses. Unlike conventional approaches that assess bone healing only in terms of total filling, this framework allows for spatially and temporally resolved investigation of how distinct tissue compartments contribute to osteoconduction. It enabled us to couple histology and immunohistochemistry with zone-specific transcriptomics, providing an integrated view of regeneration dynamics. Such a depth- and zone-resolved approach is particularly relevant for vertical ridge augmentation, an environment marked by hypoxia, limited vascularization, and mechanical stress, where bone formation is especially challenging (Elgali et al., 2016; Turri et al., 2016; Pacheco et al., 2018). Within this context, scaffold architecture plays a decisive role in directing cell migration, vascular infiltration, and matrix deposition, as pore size and interconnectivity strongly influence oxygen diffusion and osteogenic progression. Notably, more than half of the differentially expressed genes corresponded to non-coding RNAs, emphasizing the emerging regulatory influence of miRNAs and lncRNAs in osteogenic differentiation and matrix mineralization. Several of these ncRNAs are known to modulate key osteogenic regulators such as RUNX2, SP7, and components of the Wnt and BMP pathways, suggesting that scaffold-guided bone formation is partly orchestrated through post-transcriptional control (Mazziotta et al., 2021; Chen et al., 2022). Their prominence underscores a critical, yet largely unexplored, regulatory layer in biomaterial-mediated bone regeneration. Taken together, this positions our workflow as an integrative preclinical platform that connects scaffold architecture, spatial tissue organization and molecular regulation with unprecedented resolution.

### 4.2 Scaffold architecture versus material chemistry

Our findings clearly demonstrate that scaffold macroarchitecture is as decisive as material chemistry in shaping bone regeneration. Regular 3D-printed CPC scaffolds with 250 µm pores consistently outperformed both xenogenic particulates and CPC particulates of identical chemistry. This head-to-head comparison is uncommon in the literature, where scaffold macroarchitectures are often tested in separate cohorts or with confounding differences in chemistry and porosity (Lutzweiler et al., 2020; Mirkhalaf et al., 2023; Toosi et al., 2023). Clinical substitutes such as deproteinized bovine bone remain widely used, yet their randomly interconnected porosity is associated with delayed vascular penetration and slower maturation (Elgali et al., 2016; Kim et al., 2024). By directly contrasting regular and irregular arrangements of the same material, our study isolates pore geometry as the critical determinant and shows that ordered pores around 250 µm provide superior vertical ingrowth, reproducibility, and maturation. These data refine prior reports identifying the 100–400 µm range as favorable for bone ingrowth (Li et al., 2006; Yamahara et al., 2022), underscoring that smaller, ordered pores within this window optimize osteoconduction in vertical defects. Although not directly assessed in this study, it is conceivable that such architectural cues also influence mechanotransduction pathways involving Piezo1, integrin–FAK, and YAP/TAZ signaling. These mechanisms could hypothetically mediate the link between scaffold geometry, cytoskeletal tension, and osteogenic differentiation (Dupont et al., 2011). While our transcriptomic analysis highlighted strong regulation of osteogenic and ncRNA-associated pathways, the potential involvement of mechanotransduction remains to be confirmed and could be tested by introducing mechanical loading in the present model or using load-adapted systems such as calvarial defect models with vibration or ultrasound stimulation and dynamic *in vitro* bioreactors controlling shear or strain (Inoue et al., 2023; Jia et al., 2025). Future studies should aim to assess markers such as Piezo1, FAK phosphorylation, and YAP/TAZ nuclear localization to clarify how scaffold geometry and stiffness may translate into osteogenic signaling cascades. Such analyses would complement the present molecular findings and help establish a mechanistic bridge between architectural design and functional bone regeneration.

### 4.3 Autophagy as a mechanistic regulator of architecture-guided healing

Autophagy, a conserved cellular degradation and recycling process that maintains homeostasis under stress conditions, is now recognized as a central regulator of bone biology, orchestrating osteoblast differentiation, osteoclast function, and mesenchymal stem cell fate under both physiological and regenerative conditions (Yin et al., 2019; Wang et al., 2023). In challenging contexts such as osteoporosis, aging, or osseointegration around implants, modulation of autophagic flux can significantly alter outcomes (Zhang et al., 2021; Cui et al., 2023; Xu et al., 2024). Scaffold macroarchitecture, particularly pore size and interconnectivity, influences oxygen and nutrient gradients, thereby indirectly shaping autophagy signaling: macroporosity supports vascularization and reduces stress, while micro- to nano-porosity can enhance osteogenic differentiation via controlled autophagy activation (Lutzweiler et al., 2020; Zhang et al., 2021; Toosi et al., 2023). Our transcriptomic and qPCR results extend this literature by showing that autophagy-related genes (ULK1, WIPI1) are differentially regulated between 250 μm and 500 µm scaffolds, suggesting that autophagic activity is architecture-sensitive during vertical osteoconduction. Notably, direct data on autophagy dynamics in vertical augmentation remain scarce; most existing insights derive from fracture healing or systemic disease models. Our findings therefore provide novel evidence that scaffold pore size modulates autophagy in the demanding environment of vertical ridge regeneration.

### 4.4 Inflammation and immune kinetics: timing as the key variable

It is well established that a sequential immune response—initial M1 macrophage and neutrophil activity, followed by M2 macrophage–driven tissue remodeling—is essential for bone healing (Su et al., 2022; Bi et al., 2024; Gou et al., 2024). Our data are consistent with this paradigm but highlight an important nuance: scaffold macroarchitecture alters the tempo of this cascade rather than the cellular players involved. In the 250 µm condition, early pro-inflammatory cytokines (IL1β, CCL2) were upregulated, supporting angiogenesis and progenitor recruitment, while markers of NET formation (MPO, TNFα) and autophagy decreased more rapidly, suggesting faster resolution. Immunohistochemistry confirmed that M1/M2 transitions occurred deeper and earlier within the 250 µm scaffolds, which already contained mineralized bone and marrow inclusions at 4 weeks, while 500 µm scaffolds remained dominated by fibrous osteoid tissue. Thus, architecture does not merely dictate the quantity of bone but controls the kinetics of immune resolution and tissue maturation.

### 4.5 Methodological novelty and 3R compliance

Beyond biological findings, our study introduces an innovative and 3R-compliant methodological framework. By embedding two distinct pore architectures within the same scaffold and implanting them in the same animal, we generated within-animal comparisons that substantially reduce animal numbers compared to traditional parallel-group designs (Lutzweiler et al., 2020; Toosi et al., 2023). Furthermore, we integrated histology, immunohistochemistry, and laser microdissection-based transcriptomics from the same biopsy slides, enabling direct linkage between tissue organization and gene expression. While previous studies have applied laser capture microdissection (Pacheco et al., 2018) or divided tissues for separate histology and qPCR (Elgali et al., 2016; Turri et al., 2016), few have systematically combined these methods, and virtually none in the context of vertical ridge augmentation. This positions our model as a rare, integrative preclinical platform that maximizes data yield while adhering to reduction and refinement principles.

### 4.6 Limitations

Despite these strengths, several limitations should be acknowledged.

First, differences in regenerative capacity across species must be considered. However, while not a direct analogue to human bone healing, rabbit bone shares several structural and metabolic similarities with human bone structure and metabolism (Pearce et al., 2007). Rabbit represents approximately 80% of all published reactor-based calvarial studies. In our own previous work using the same model, we observed bone formation trends that were noticeably lower than those typically reported in larger-animal models such as sheep (Carrel et al., 2016a; Marger et al., 2019; Durual et al., 2021; Marger et al., 2022). Likewise, other studies performed in rabbits or using bovine particulates consistently describe similarly modest levels of bone regeneration (Tamimi et al., 2006; Kim et al., 2010). Together, these observations confirm that the rabbit calvarial reactor model yields conservative, rather than exaggerated, regenerative outcomes. Its ability to host four reactors simultaneously enables rigorous intra-animal comparisons and high-resolution mechanistic analyses, which was central to the goals of the present study. Therefore, we position this model as a sensitive comparative system rather than a direct predictor of human outcomes.

Second, the use of a PEEK cap may be a bias compared to clinical practice where resorbable membranes are often used. In this experimental context, PEEK was intentionally chosen to ensure rigid space maintenance, geometric stability, and reproducibility across reactors, all of which are essential for mechanistic evaluation of scaffold macroarchitecture. Across several studies in which our group has employed this identical PEEK reactor configuration, we have never observed any detrimental effect of either the cylinder or the cap on osteoconduction, tissue behavior, or bone formation (Marger et al., 2019; Durual et al., 2021; Marger et al., 2022). PEEK has consistently behaved as an inert, non-interfering structural support. Nonetheless, we agree that future studies may investigate the same architectural variables under resorbable membranes to more closely reflect clinical workflows.

Finally, the acellular scaffold design isolates the architectural contribution but does not capture interactions relevant to emerging cell-laden bioprinting approaches, which may constitute a valuable direction for future investigations.

## 5 Conclusion

In summary, our results highlight that scaffold pore architecture is as decisive as chemistry in determining bone regeneration quality. A 250 µm ordered pore design accelerates immune resolution, enhances vertical osteoconduction up to 5 mm, and engages autophagy and inflammatory pathways in a pore-size–dependent manner. The dynamic bone growth model provides a unique analytical framework to resolve spatial and molecular events through depth-resolved mapping of migrating zones, thereby revealing the vertical choreography of tissue regeneration. Finally, by integrating histology, immunohistochemistry, and transcriptomics within a single workflow, our study establishes an integrative preclinical platform that both advances mechanistic insight and aligns with 3R principles of experimental refinement.

## Data Availability

Datasets are available on request: The raw data supporting the conclusions of this article will be made available by the authors, without undue reservation. qRT-PCR raw data may be find at https://doi.org/10.5281/zenodo.18471465.
